# Lanthanum regulates the reactive oxygen species in the roots of rice seedlings

**DOI:** 10.1038/srep31860

**Published:** 2016-08-22

**Authors:** Dongwu Liu, Shengnan Zheng, Xue Wang

**Affiliations:** 1 School of Life Sciences, Shandong University of Technology, Zibo, Shandong, 255049, China

## Abstract

In this study, the effects of La^3+^ on the reactive oxygen species (ROS) and antioxidant metabolism were studied in the roots of rice (*Oryza sativa* L. cv Shengdao 16) exposed to increasing concentrations of La^3+^ (0.05, 0.1, 0.5, 1.0, and 1.5 mM). The level of hydrogen peroxide, superoxide anion, and malondialdehyde was increased by 0.5, 1.0 and 1.5 mM La^3+^, and the activity of catalase and peroxidase was increased by 0.05 and 0.1 mM La^3+^. However, La^3+^ treatments stimulated superoxide dismutase activity in the roots of rice seedlings at all tested concentrations. In addition, the probe 2′,7′-dichlorofluorescein diacetate (H_2_DCF-DA) was used to investigate the instantaneous change of ROS in the root cells with the laser-scanning confocal microscopy. The result indicated that ROS level was declined after treated with 0.05 mM La^3+^. The results showed that the appropriate concentration of La^3+^ decreased the level of ROS, and hormetic effects on the antioxidant metabolism were found in the roots of rice exposed to 0.05, 0.1, 0.5, 1.0, and 1.5 mM La^3+^.

The rare earth elements (REEs), which have the similar chemical properties, include a group of 17 trivalent metallic elements. In China, REEs enriched fertilizers have been used since the 1970s^1^. REEs enter into the mesophyll cells via apoplast and symplast channels or plasmodesmata^2^,^3^. It has been observed that the appropriate amount of REEs has positive effects on seed germination, harvest quality, chlorophyll content, photosynthetic rate, plant biomass, and root development^4^,^5^,^6^. The growth of tobacco seedlings and light reactions of photosynthesis were stimulated by the suitable concentration of trivalent lanthanum (La^3+^)^7^. However, the results of field trials and laboratory studies on La^3+^ are still contradictory^8^,^9^,^10^.

Reactive oxygen species (ROS) mainly include hydrogen peroxide (H_2_O_2_), singlet oxygen (^1^O_2_), superoxide anion 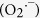, and hydroxyl radical (OH^·^). In the plant cells, ROS could act as a signal molecule and trigger a series of cellular responses^11^,^12^. It is known that ROS are toxic to plants if the accumulation levels of ROS exceed the detoxification capacity of the plant tissues. However, there are various mechanisms to cope with oxidative damage in plants. The enzymatic antioxidants, including superoxide dismutase (SOD, EC.1.15.1.1), ascorbate peroxidase (APX, EC 1.11.1.11), glutathione-S-transferase (GST, EC. 2.5.1.18), catalase (CAT, EC.1.11.1.6), and glutathione reductase (GR, EC 1.6.4.2), are involved in protecting plant cells from oxidative damage by scavenging ROS^13^,^14^,^15^.

Hormesis is defined as a phenomenon that low doses of an otherwise harmful agent could result in stimulatory or beneficial effects^16^. Accurate description of hormetic dose-response is important for the determination of the efficacy and hazards of La^3+^. In this study, the instantaneous change of ROS was investigated in the root cells treated by La^3+^ with the laser-scanning confocal microscopy. Furthermore, the hormetic effects of La^3+^ on the defense system were investigated in the rice seedlings of *Oryza sativa* L. (cv Shengdao 16).

## Results

### Effect of La^3+^ on the levels of MDA, H_2_O_2_, 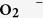 and soluble protein

**Figure 1:**
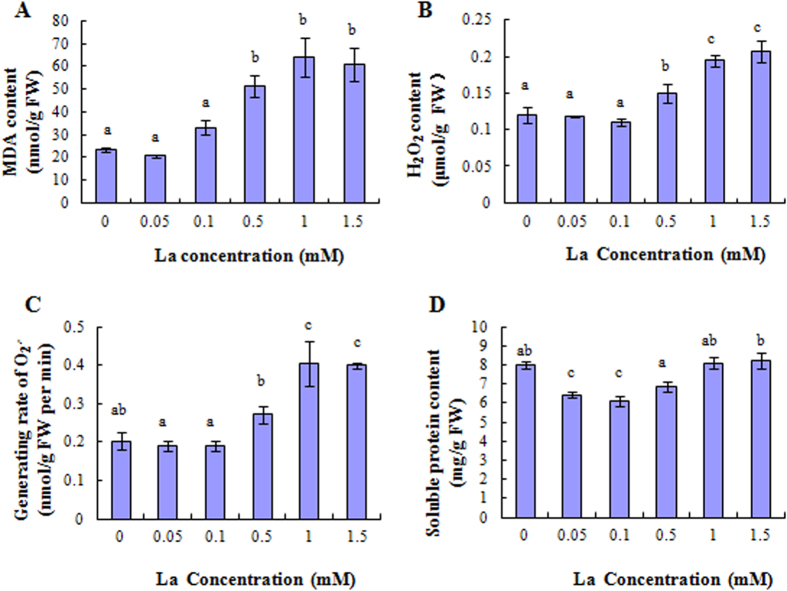
Effect of La^3+^on MDA, H_2_O_2_, 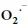, and soluble protein level in the root of rice. **(A)** Effect of La^3+^ on the level of MDA. **(B)** Effect of La^3+^ on the H_2_O_2_. **(C)** Effect of La^3+^ on the level of 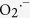. **(D)** Effect of La^3+^ on the level of soluble protein. Values represent means ± SE (n = 3). Different letters indicate significant differences (*P* < 0.05) according to tukey’s test.

The level of MDA and H_2_O_2_ was significantly increased by increasing solution La concentrations from 0.5 to 1.5 mM (Fig. 1A,B), while the level of 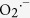 was significantly increased by 1.0 and 1.5 mM La^3+^ (Fig. 1C). However, the level of H_2_O_2_ and 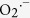 was not significantly decreased by 0.05 and 0.1 mM La^3+^ (Fig. 1B,C). Moreover, The soluble protein content was significantly decreased by 0.05 and 0.1 mM La^3+^ (Fig. 1D), but no effects was observed by increasing solution La concentrations from 0.5 to 1.5 mM (Fig. 1D).

### Effect of La on the instantaneous change of ROS in the root cells

**Figure 2:**
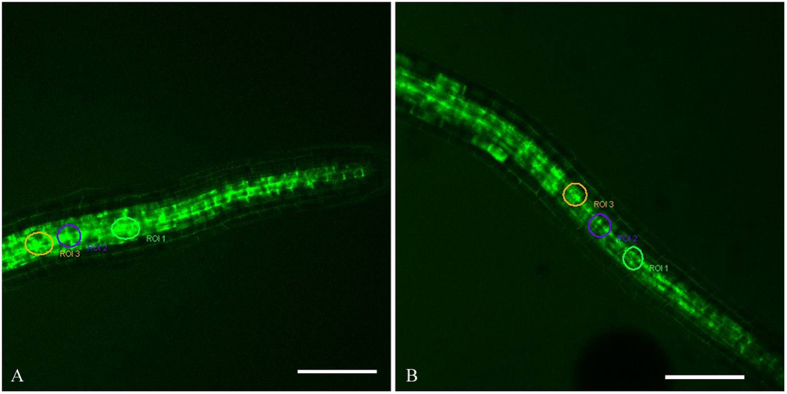
The regions of interest (ROI 1–3) in the root treated with MES-KCl buffer or 0.05 mM La^3+^. The root cells were labeled with H_2_DCF-DA and ROS (green) were imaged. The regions of interest (ROI 1–3) were circled with Leica Confocal Software. **(A)** The regions of interest (ROI 1–3) in the root treated with MES-KCl buffer. **(B)** The regions of interest (ROI 1–3) in the root treated with 0.05 mM La^3+^. Bar: 75 μm.

**Figure 3:**
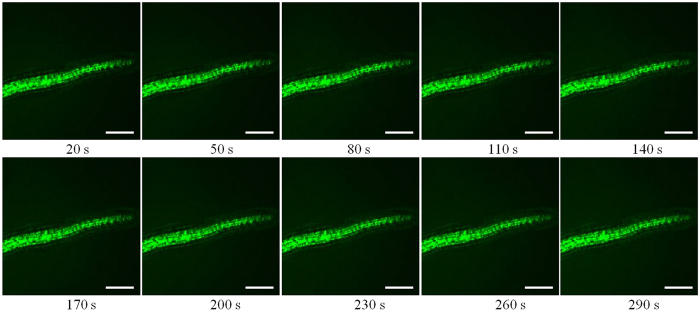
Effects of MES-KCl buffer on ROS in the root cells. The root cells were labeled with H_2_DCF-DA and ROS (green) were imaged. The laser-scanning mode was XYT, and the time interval between two sections was 10.0 s. Thirty optical sections were acquired with the laser-scanning confocal microscope, and the MES-KCl buffer was added at about 40 s. Ten images at different time were selected in the figures. Bar: 75 μm.

**Figure 4:**
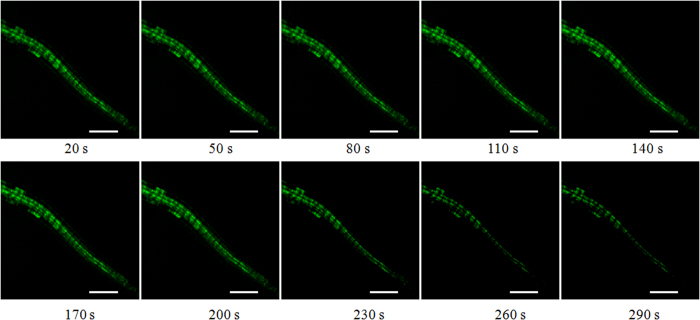
Effects of 0.05 mM La^3+^ on ROS in the root cells. The root cells were labeled with H_2_DCF-DA and ROS (green) were imaged. The laser-scanning mode was XYT, and the time interval between two sections was 10.0 s. Thirty optical sections were acquired with the laser-scanning confocal microscope, and 0.05 mM La^3+^ was added at about 40 s. Ten images at different time were selected in the figures. Bar: 75 μm.

**Figure 5:**
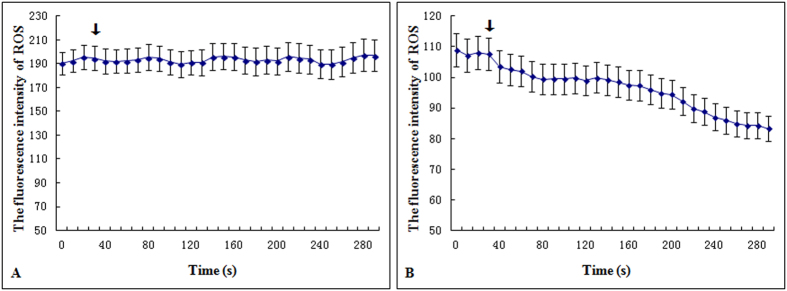
Effect of MES-KCl buffer or 0.05 mM La^3+^ on ROS in the root cells. (**A**) Effect of MES-KCl buffer on ROS in the root cells. (**B**) Effects of 0.05 mM La^3+^ on ROS in the root cells. The arrow indicates the time that MES-KCl buffer or 0.05 mM La^3+^ was added at about 40 s. Values represent means ± SE (n = 3).

The fluorescence of ROS (green) was imaged in the cells of root and the regions of interest (ROI 1–3) were circled with Leica Confocal Software (Fig. 2). The fluorescence intensity of ROS in the root cells was processed and quantified with Leica Confocal Software. Compared to the control, the level of ROS was declined in the root cells treated with 0.05 mM La^3+^ (Figs 3,4 and 5).

### Effect of La^3+^ on SOD, POD and CAT activity, and La^3+^ accumulation

**Figure 6:**
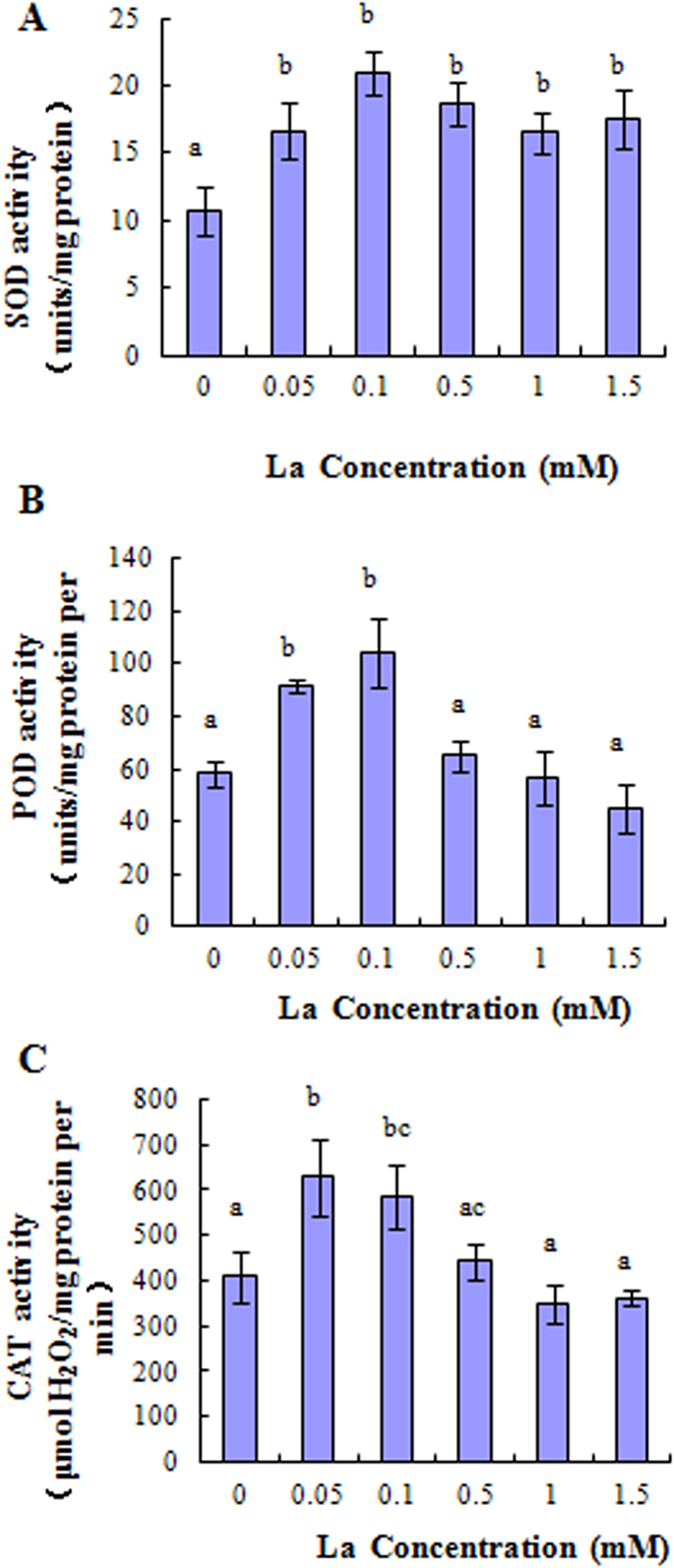
Effect of La^3+^ on the activity of SOD, POD, and CAT in the root of rice. **(A)** Effect of La^3+^ on the activity of SOD. **(B)** Effect of La^3+^ on the activity of POD. **(C)** Effect of La^3+^ on the activity of CAT. Values represent means ± SE (n = 3). Different letters indicate significant differences (*P* < 0.05) according to tukey’s test.

**Figure 7:**
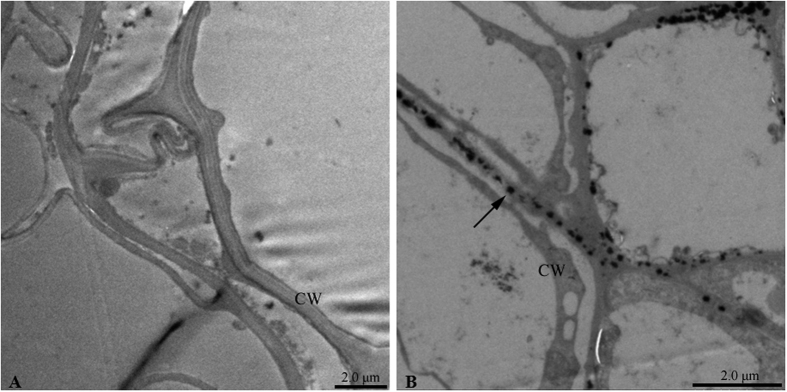
The subcellular distribution of La in the root. (**A**) Control, showing no La particle deposited in the cell wall (CW); (**B**) root cell treated with 1.0 mM La^3+^, showing La particles located in the CW. The arrow indicates La particles.

Antioxidant assays were performed on the roots of seedlings collected after 13 days treatment with La^3+^. Compared to the control, the activity of SOD was significantly increased in roots after La^3+^ treatments (Fig. 6A). The activity of POD and CAT was significantly increased by 0.05 and 0.1 mM La^3+^ (Fig. 6B), However, POD and CAT activity was unaffected by increasing solution La concentrations from 0.5 to 1.5 mM (Fig. 6B). In addition, the highest increase of SOD and POD activity was observed at 0.1 mM La^3+^ (Fig. 6A,B), but the highest CAT activity was at 0.05 mM La^3+^ (Fig. 6C). Moreover, La^3+^ particles were located in the cell wall with the technique of transmission electron microscope (Fig. 7).

## Discussion

In this study, the level of H_2_O_2_, 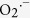, and MDA was significantly induced by 1.0 and 1.5 mM La^3+^, but not significantly inhibited by 0.05 and 0.1 mM La^3+^. It has been reported that low concentrations of La^3+^ alleviated the oxidative damage induced by UV-B radiation through inhibiting the production of H_2_O_2_, 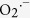, and MDA^17^. The results of Wang *et al*. reported that La^3+^ protected soybean plants from oxidative stress by improving the defense system of plants^18^. However, the level of H_2_O_2_, 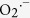, and MDA was not significantly affected by low concentrations of La^3+^ in this study. The reason may be that the concentrations of La^3+^ were different from the previous studies. In addition, the increase of antioxidants induced by lanthanum nitrate treatment at low concentrations has been reported in the aged *Oryza sativa* L., and the production of ROS was successfully controlled by the antioxidant stimulation^19^. In this study, it was found that the activity of CAT and POD was induced by 0.05 and 1.0 mM La^3+^, but not affected by 0.5, 1.0, and 1.5 mM La^3+^. Additionally, La^3+^ treatments stimulated SOD activity in the roots of rice seedlings at all tested concentrations. The results indicated that some protective enzymes were activated by 0.05 and 1.0 mM La^3+^. The formation of H_2_O_2_ induced by higher concentrations of La^3+^ may be associated with the increased activity of SOD for 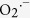 conversion.

The concentration of REEs is not consistent in the previous studies. The results of Ippolito *et al*. showed that 5.0 mM La^3+^ did not cause either visible symptoms on plants or significant effects on ROS production, chlorophyll content, and lipid peroxidation in common duckweed^20^. Diatloff *et al*. found that La^3+^ induced the growth of corn and mungbean when the concentration was below 0.2 μM^9^. However, Chen *et al*. reported 60 mM La^3+^ significantly promoted the growth of callus^21^. In this study, it appears that the higher concentration of La^3+^ induces oxidative stress in the root cells, and the appropriate concentration of La^3+^ on growth may be related with the different plant species.

REEs can be absorbed into plant cells, which is the basis for interpreting biochemical effects of REEs on plant cells^22^. In this study, the distribution of ROS is not consistent in the root cells. There are more ROS in some root cells, and less ROS in the other root cells. The reason may be that the root cells accumulate the different amount of La^3+^, which leads to the different ROS distribution in the root cells. Wang *et al*. reported that La^3+^ protected soybean plants from oxidative stress by directly reacting with ROS^18^. Our results also show that the appropriate concentration of La^3+^ could decrease the level of ROS with confocal microscopy.

In this study, it was found that the effect of La^3+^ on the antioxidant metabolism was related to the concentration of La^3+^. Once the accumulation of La^3+^ exceeds the detoxification capacity of the plant tissues, it will be toxic to plant cells^23^. In the study, this threshold was reached at 0.5 mM La^3+^ in the nutrient solution. Hormetic effects generally show two kinds of trends, including the low-dose-stimulation and the high-dose-inhibition effects^16^,^24^. In this study, the hormetic effects on the antioxidant metabolism were also observed in the roots of rice treated with low and high concentrations of La^3+^.

In summary, the probe H_2_DCF-DA was used to investigate the instantaneous change of ROS with the laser-scanning confocal microscopy. It showed that the appropriate concentration of La^3+^ decreased the level of ROS in the root cells. Hormetic effects on the antioxidant metabolism were found in the roots of rice exposed to increasing concentrations of La^3+^ (0.05, 0.1, 0.5, 1.0, and 1.5 mM).

## Methods

### Plant material and plant growth

Seeds of rice (*O. sativa* L. cv Shengdao 16) were sterilized by soaking in 75% alcohol for 1 min, in 0.1% mercury chloride for 15 min, and in 1.0% sodium hypochlorite for 20 min. Then the seeds were rinsed five times with sterilized water, and germinated in half-strength Murashige and Skoog agar medium at pH 5.8 (0.75 mM MgSO_4_, 10 mM NH_4_NO_3_, 9.4 mM KNO_3_, 0.625mM KCl, 1.5 mM CaCl_2_, 2.5 μM KI, 50 μM H_3_BO_3_, 50 μM FeSO_4_, 50 μM MnSO_4_, 15 μM ZnSO_4_, 0.05 μM CuSO_4_, 0.05 μM CoCl_2_, 0.5 μM Na_2_MoO_4_, 50 μM Na_2_H_2_EDTA, 0.15 μM thiamine, 1.2 μM pyridoxine, 2 μM nicotinic acid, 275 μM inositol, 0.56% agar, 3.0% sucrose, 0.05% Mes). Subsequently, La(NO_3_)_3_ was added to the basal medium before autoclaving to obtain La^3+^ concentrations of 0, 0.05, 0.1, 0.5, 1.0, and 1.5 mM. Plants were grown 13 days in the different concentration of La^3+^ at 25.0 ± 2 °C using a 14/10 h light/dark cycle under a light intensity of 200 μmol m^−2^ s^−1^ in a growth chamber.

### Assay antioxidant enzyme activities

Plants were collected and the fresh weight (FW) of roots was determined in 13 day-old seedlings. The roots biomass (0.5 g in fresh mass) was homogenized under ice-cold conditions in 5.0 ml of extraction buffer containing 50 mM phosphate buffer (pH 7.5), 1.0% polyvinylpyrrolidone (PVP), 0.5% Triton X-100 and 1 mM EDTA, centrifuged at 10,000 × g at 4 °C for 20 min to remove particulate plant debris. The supernatant was used for the assay of antioxidant enzyme activities. The SOD activity was determined at 550 nm, and one unit of SOD activity was the amount of enzyme that inhibits 50% nitrite formation^25^. The CAT activity was assayed at 405 nm based on the principle that H_2_O_2_ could react with ammonium molybdate and form a stable complex, and one unit of CAT decomposes 1.0 μM H_2_O_2_ per minute^26^. The POD activity was determined by an increase in absorbance at 470 nm during the oxidation of guaiacol^27^.

### Assay the levels of soluble protein, H_2_O_2_, 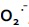 , and MDA

The plant samples were obtained as the assay of antioxidant enzyme activities. The same supernatant was used for the determination of soluble proteins, H_2_O_2_, 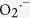, and malondialdehyde (MDA). The content of H_2_O_2_ was determined at 405 nm based on the principle that H_2_O_2_ could form a stable complex with ammonium molybdate^26^. The soluble proteins were assayed with bovine serum albomin as standard protein according to the Bradford method^28^. The level of 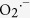 was determined at 530 nm according to procedures described by (Wang and Luo)^29^, and the level of MDA was measured for it could react with thiobarbituric acid^30^.

### Effect of La on the instantaneous change of ROS in the root cells

The production of ROS in the roots was visualized with 2′,7′-dichlorofluorescein diacetate (H_2_DCF-DA, Molecular Probes). For assessment of ROS, the rice roots were immersed in 1.5 ml of 10 μM DCFH-DA (dissolved in MES-KCl buffer, KCl 50 mM, MES 10 mM, pH 5.5) for 2 hr at 20 °C. Then the roots were rinsed with MES-KCl buffer (KCl 50 mM, MES 10 mM, pH 5.5) to remove DCFH-DA solution from the surface of roots.

A laser-scanning confocal microscope (Leica TCS SP2, Germany) with an argon-ion laser as the excitation source at 488 nm was used to view the sites of ROS in the root cells. The laser-scanning mode was XYt, 30 optical sections were acquired, and the time interval between two sections was 10.0 s. To investigate the instantaneous change of ROS in the root, MES-KCl buffer or 0.05 mM La^3+^ was added into 35 mM petri dish after four optical sections were scanned, respectively. Then the regions of interest (ROI 1–3) were circled with Leica Confocal Software, and the fluorescence intensity of ROI was acquired. Data were processed with a Leica TCS Image Browser, and transferred to Adobe Photoshop 6.0 for preparation of figures.

### Transmission electron microscope analysis

The roots were fixed for 2 h at 4 °C in 2.5% (v/v) glutaraldehyde and 0.1 M phosphate buffer solution (pH 7.3), and postfixed in 1% (w/v) aqueous osmium tetraoxide for 2 h. Samples were dehydrated in a 50–100% ethanol series and embedded in Epon 812 resin. Ultra-thin sections of 70 nm thickness were cut with an Ultracut Eultramicrotome (Leica, Germany) and stained with uranyl acetate and lead citrate. Then the subcellular distribution of La was detected with a Hitachi H-600 transmission electron microscope (TEM).

### Statistical analyses

The assays for oxidative stress and the soluble protein were carried out in three different experiments, and results are expressed as mean ± standard error (SE). Statistical comparisons were done with one-way ANOVA using SPSS 16.0 for Windows (SPSS Inc., Chicago, USA). Tukey test was performed for post hoc comparisons when the difference was significant (*P* < 0.05).

## Additional Information

**How to cite this article**: Liu, D. *et al*. Lanthanum regulates the reactive oxygen species in the roots of rice seedlings. *Sci. Rep*. **6**, 31860; doi: 10.1038/srep31860 (2016).
